# Comparative genomic and resistome analysis of *Citrobacter koseri*: first genome report from Egypt

**DOI:** 10.1186/s12866-026-05726-w

**Published:** 2026-11-05

**Authors:** Samira M. Hamed, Manal M. Darwish, Mohamed Samir, Reham Monir, Ahmed Al Taweel, Ayat I. Ghanem, Ihab N. Hanna, Aya H. Hefnawy, Nada S. Gebreel, Noha M. Soltan, Yara H. Aboudewan, Mai A. Amer

**Affiliations:** 1 Microbiology and Immunology Department, October University for Modern Sciences and Arts (MSA), Giza, Egypt; 2 Medical Microbiology and Immunology Department, Faculty of Medicine, Ain Shams University, Cairo, Egypt; 3 School of Science, Faculty of Engineering and Science, University of Greenwich (Medway campus, Kent), London and Southeast (LASE) University Group, London, UK; 4 Department of Zoonoses, Faculty of Veterinary Medicine, Zagazig University, Zagazig, Egypt; 5 National Institute of Diabetes and Endocrinology (NIDE), Cairo, Egypt; 6 PharmD Students Program, Faculty of Pharmacy, October University for Modern Sciences and Arts (MSA), Giza, Egypt

**Keywords:** *Citrobacter Koseri*, Genomics, Antimicrobial resistance, Diabetic foot ulcer (DFU), Bioinformatics, virulence, *mcr*, KPC

## Abstract

**Background:**

*Citrobacter koseri* is increasingly implicated in healthcare- and community-associated infections, with multidrug-resistant (MDR) strains posing a growing clinical concern. However, its population structure and resistome dynamics remain incompletely understood. Here, we report the first draft genome sequence of an MDR *C. koseri* isolate (DFU32) from Egypt, recovered from a patient with a diabetic foot ulcer (DFU). We also present a comparative genomic and resistome analysis of 518 publicly available *C. koseri* genomes available at the time of the study.

**Results:**

The isolate exhibited resistance to multiple antimicrobial classes, including β-lactams, aminoglycosides, fluoroquinolones, and sulfonamides, as well as tigecycline and colistin. MLST assigned DFU32 to sequence type (ST) 1172. Genomic analysis identified 21 antimicrobial resistance (AMR) genes, including multiple β-lactamases (*bla*_CKO_, *bla*_TEM_, *bla*_OXA−9_, *bla*_CTX−M−15_, and *bla*_CMY−2_) and aminoglycoside resistance determinants, including *armA*. Colistin resistance was associated with the presence of a PhoQ V381I substitution. Comparative analysis of 518 publicly available genomes revealed 140 unique AMR genes across the species, with 73.6% of genomes carrying acquired resistance determinants. The population comprised two major lineages, *bla*_CKO_ and *bla*_MAL_, with the MAL lineage, particularly MAL-2, carrying significantly more AMR genes. Carbapenemase genes were identified in 29.1% of genomes and were distributed across nearly half of the detected STs, with *bla*_KPC_ variants predominating. Mobilized colistin resistance (*mcr*) genes were detected in 2.5% of genomes. Both carbapenemase and *mcr* genes were predominantly located on plasmid-associated contigs. Notably, chromosomal integration events were observed. These included *mcr-9.1*, which was associated with IS26 and IS903B, and *bla*_KPC−2_, which was embedded within an IS26-bracketed transposon (IS*26*–*recA*–IS*Kpn27*–*bla*_KPC−2_–IS*Kpn6*–IS*26*) in novel STs. These findings underscore the genomic plasticity of *C. koseri* and the potential for stable, plasmid-independent maintenance of colistin and carbapenem resistance. Among the identified STs, ST690 displayed a particularly high resistance gene load, including carbapenemase and *mcr* genes, suggesting that it may represent a high-risk lineage.

**Conclusion:**

This study provides the first genome-based characterization of an MDR *C. koseri* isolate from Egypt. The results highlight the potential genetic mobility and global spread of AMR determinants in this emerging pathogen. Notably, the studied isolate was recovered from DFU, underscoring the increasing clinical relevance of *C. koseri* in chronic wound infections.

**Supplementary Information:**

The online version contains supplementary material available at https://doi.org/10.1186/s12866-026-05726-w.

## Introduction

*Citrobacter koseri* (formerly *Citrobacter diversus*) is a Gram-negative, rod-shaped, facultative anaerobic bacterium that belongs to the genus *Citrobacter*. It is commonly found in the human gastrointestinal tract as part of the normal microbiota and is also present in soil, water, and various food sources. Transmission of *C. koseri* can occur via contaminated food or water, the fecal-oral route, or direct person-to-person contact, especially in healthcare settings [1]. Although it is regarded as a low-virulence opportunistic pathogen, *C. koseri* can cause severe infections, particularly in immunocompromised individuals, neonates, and children [2–4]. Reports of complicated *C. koseri* infections in adolescents and adults have also been documented [5, 6].

Recent surveillance studies in India indicate a marked increase in *Citrobacter*-associated bacteremia, rising from 12% in 2016 to 24% in 2022, with *C. koseri* accounting for approximately 72% of all *Citrobacter* bacteremia cases [7]. The pathogen causes particularly severe disease in neonatal and pediatric populations, where it is linked to invasive infections such as sepsis, meningitis, and brain abscesses. These infections have an estimated mortality rate of 33%, and nearly half of survivors develop long-term neurological complications [8]. Recurrent outbreaks in neonatal intensive care units further highlight its potential for hospital-acquired transmission among highly vulnerable populations [9].

Mortality associated with *Citrobacter* infections appears to be more strongly linked to the antibiotic resistance profile of the isolate than to the specific *Citrobacter* species involved [10]. *C. koseri* is intrinsically resistant to aminopenicillins and carboxypenicillins. Infections caused by this species are commonly treated with antibiotics such as cephalosporins, carbapenems, aminoglycosides, quinolones, and chloramphenicol [11]. Over time, *C. koseri* has shifted from being largely susceptible to frontline antibiotics to showing increasingly complex resistance patterns. Recent strains often carry a combination of mobile genetic elements and chromosomal mutations that reduce the effectiveness of β-lactams, including extended-spectrum cephalosporins and carbapenems [12, 13]. More recently, resistance has expanded to include last-resort agents such as colistin, underscoring its adaptive capacity and the growing therapeutic challenges associated with its management. Therefore, treatment decisions should be guided by susceptibility testing and resistome analysis to ensure efficacy. Collectively, the growing body of evidence from 2021 to 2025 indicates that *C. koseri* is evolving from a traditionally opportunistic organism into a clinically important healthcare-associated pathogen with increasing multidrug resistance and expanding epidemiological significance.

Considering the rising incidence and clinical significance of *C. koseri* infections, this study aimed to investigate the genomic landscape of a multidrug-resistant (MDR) *C. koseri* isolate, representing the first draft genome of this species reported from Egypt, and to perform a comprehensive resistome comparison with all 518 complete and draft *C. koseri* genomes available in the National Center for Biotechnology Information (NCBI) genomic database at the time of analysis.

## Materials and methods

### Isolation, identification, and antimicrobial susceptibility testing of *C. koseri* (DFU32)

The *Citrobacter koseri* strain DFU32 sequenced in the current study was originally isolated from an infected diabetic foot ulcer (DFU) of a 48-year-old diabetic female patient admitted in March 2024 to the National Institute for Diabetes and Endocrinology in Cairo, Egypt. The isolates were submitted to the microbiology laboratory for routine microbiological identification and antimicrobial susceptibility testing as part of the standard clinical care for patients with infected DFUs. Identification was performed using conventional microbiological techniques and was confirmed using the VITEK^®^ 2 automated system (bioMérieux, Lyon, France) with Gram-negative identification (GN ID) cards.

Phenotypic antimicrobial susceptibility testing of strain DFU32 was conducted using the Kirby-Bauer disc diffusion assay with commercially sourced antibiotic discs (Oxoid, UK). The tested antibiotics and their respective concentrations were as follows: amoxicillin/clavulanic acid (20/10 µg), piperacillin/tazobactam (100/10 µg), cefoxitin (30 µg), ceftriaxone (30 µg), cefotaxime (30 µg), cefepime (30 µg), aztreonam (30 µg), meropenem (10 µg), gentamicin (10 µg), amikacin (30 µg), ciprofloxacin (5 µg), tetracycline (30 µg), tigecycline (15 µg), chloramphenicol (30 µg), nitrofurantoin (300 µg), fosfomycin (200 µg), and trimethoprim/sulfamethoxazole (1.25/23.75 µg). Due to the poor and unpredictable diffusion of colistin in agar, susceptibility testing was performed using broth microdilution (0.125–128 µg/mL, Sigma-Aldrich, USA), as recommended by Matuschek et al., [14]. Interpretation of susceptibility results followed the guidelines established by the Clinical and Laboratory Standards Institute [15], except for tigecycline, which was evaluated using EUCAST v14.0 breakpoints for *Enterobacterales* [16]. For quality control, *Escherichia coli* ATCC 25,922 was included. The MDR phenotype was determined according to the criteria described by Magiorakos et al., [17], which define MDR as non-susceptibility to at least one antimicrobial agent in three or more antimicrobial classes.

### DNA extraction and whole genome sequencing of *C. Koseri* (DFU32)

Genomic DNA extraction of the isolate *C. koseri* DFU32 was performed using QIAamp DNA Kits (Qiagen, Hilden, Germany) in accordance with the manufacturer’s protocol. Whole-genome sequencing (WGS) and library preparation were subsequently conducted by BGI Tech Solutions Hong Kong Co., Ltd., China, employing DNBseq™ short-read sequencing technology. Sequencing was performed using paired-end 150 bp reads (PE150).

### Draft genome assembly, annotation, and analysis of *C. Koseri* (DFU32)

Raw sequencing reads underwent quality control and adapter trimming during pre-processing using BGI’s SOAPnuke software [18]. For genome reconstruction, high-quality paired-end short reads were subjected to de novo assembly using Unicycler v0.4.8 [19]. Assembly was performed using 12 threads (-t 12), with a minimum FASTA sequence length of 300 bp (--min_fasta_length 300). The paired-end short reads were provided using the --short1 and --short2 options. No long-read data were used for the assembly. Post-assembly quality metrics were assessed using QUAST v5.2.0 [20] and genome completeness was evaluated using CheckM2 [21]. Plasmid content was subsequently analysed using mob-recon v3.1.9, a component of the MOB-suite package [22] to reconstruct and classify plasmid-associated sequences from the draft genome assembly.

The draft genome was subsequently annotated using the NCBI Prokaryotic Genome Annotation Pipeline (PGAP) V6.10 [23]. Genome-based identification was conducted as described before [24]. First, by analyzing 16 S rRNA gene sequences using the EzBioCloud server [25]. Subsequently, the Average Nucleotide Identity (ANI) between genomes was calculated using JSpeciesWS [26] while digital DNA-DNA hybridization (dDDH) values were computed through the Type Strain Genome Server (TYGS) [27].

### Virulome analysis of DFU32

The draft genome of DFU32 was screened for putative virulence-associated genes (VGs) using three complementary homology-based approaches: VFanalyzer [28], ABRicate v1.4.0, and VirulenceFinder v3.2.1 [29]. Because *Citrobacter* was not available among the supported genera in VFanalyzer, the genome was analysed separately using *Escherichia*,* Salmonella*,* Shigella*,* Yersinia*, and *Klebsiella* as reference genera, and the resulting outputs were combined. ABRicate was used to screen the genome against the Virulence Factor Database (VFDB; database update: 3 April 2026) [30], using thresholds of ≥ 80% sequence identity and ≥ 80% coverage. VirulenceFinder was run using its default thresholds of ≥ 90% sequence identity and ≥ 60% coverage. Because *C. koseri* was not among the supported species in VirulenceFinder, the “all databases (default)” option was selected. The outputs from the three approaches were subsequently compared to identify shared and tool-specific VGs.

### MLST and Phylogenetic analysis of DFU32 and global *C. Koseri* strains

A collection of 518 draft (*n* = 482) and complete (*n* = 36) *C. koseri* genomes were retrieved from the NCBI genome database (accessed on 20 July 2025) for subsequent analysis. Metadata for all genomes are summarized in Supplementary Table 1. Multilocus sequence type (MLST) of DFU32 and the retrieved *C. koseri* strains was conducted using FastMLST v0.0.16 [31] which performs local allele searches by running BLASTn against the regularly updated allele datasets from the PubMLST database [32]. The MLST scheme for *Citrobacter* species, available on PubMLST, was originally developed by Bai et al., [33] and is based on the sequences of seven housekeeping genes, including *aspC*, *clpX*, *fadD*, *mdh*, *arcA*, *dnaG*, and *lysP*.

Single-nucleotide polymorphism (SNP)–based phylogenetic analysis was conducted using the Snippy pipeline v4.0.2 [34] with the complete genome sequence of *C. koseri* ATCC BAA-895 (GenBank accession: GCA_000018045.1) used as the reference. Draft and complete genome assemblies of all isolates (*n* = 519) were analysed using Snippy in contig mode (--contigs) under default parameters. In this mode, genome assemblies are shredded into synthetic reads and aligned to the reference genome using the integrated BWA-MEM mapper, followed by variant calling with FreeBayes to identify SNPs. Core genome SNPs shared among all isolates were extracted using Snippy-core, generating a concatenated alignment of high-quality variant sites.

The core genome SNP alignment generated using Snippy was subsequently analysed with Gubbins v3.4.3 [35] to identify and account for homologous recombination. Gubbins was run iteratively, using IQ-TREE v2.4.0 [36] and RAxML-NG v2.0.2 [37] as tree builder (using commands: --first-tree-builder iqtree-fast and --tree-builder raxmlng). Recombination events were characterised based on Gubbins predictions, including recombination blocks (genomic regions inferred to have been introduced through homologous recombination, based on clusters of SNPs showing patterns inconsistent with the expected clonal evolutionary history), SNPs per recombination event, and the r/m ratio (the relative contribution of recombination to mutations). Pairwise SNP distances were subsequently calculated from the recombination-filtered alignment generated by Gubbins using snp-dists v1.2.0, available at: https://github.com/tseemann/snp-dists. The resulting phylogenetic tree was visualized, annotated, and edited using the Interactive Tree Of Life (iTOL) online tool [38]. Country-level classification of all public genomes was not available.

### Resistome analysis of the species *C. koseri* and comparative resistome analysis of DFU32

Antimicrobial resistance (AMR) genes present in all *C. koseri* genomes deposited in the NCBI database were obtained from the NCBI National Database of Antibiotic Resistant Organisms (NDARO) [39], specifically from its Pathogen Detection Microbial Browser for Identification of Genetic and Genomic Elements (MicroBIGG-E). MicroBIGG-E was searched for *Citrobacter koseri* and AMR category to identify resistance genes. For DFU32 and genomes not included in the MicroBIGG-E database (*n* = 27), resistome profiling was conducted manually using AMRFinderPlus 4.0 [39] employing matched threshold to that used by MicroBIGG-E.

The genetic environments of the resistance genes were visualized using Proksee web interface, available at: https://proksee.ca/ [40]. Replicon typing of plasmids harbouring carbapenemase- or *mcr*-encoding genes was performed using ABRicate (v1.2.0) against the PlasmidFinder database. Replicons were identified using the same minimum identity and coverage thresholds employed by PlasmidFinder 2.1 online tool (95% and 80%, respectively) [41].

### Statistical analysis

All statistical analyses were performed using IBM SPSS Statistics for Windows, version 26.0 (IBM Corp., Armonk, NY, USA). Measures of central tendency were expressed as medians and means. All tests of significance were two-tailed, with statistical significance defined at *P* ≤ 0.05. The normality of continuous variables was assessed using the Shapiro–Wilk test. As the data did not follow a Gaussian distribution, comparisons between groups were performed using the Mann–Whitney U test.

## Results

### Phenotypic antimicrobial susceptibility profile of DFU32

DFU32 exhibited an MDR phenotype. The isolate was non-susceptible to amoxicillin/clavulanic acid and extended-spectrum cephalosporins (cefoxitin, ceftazidime, cefotaxime, and cefepime), as well as to aminoglycosides (gentamicin and amikacin), tetracycline, tigecycline, chloramphenicol, nitrofurantoin, and trimethoprim/sulfamethoxazole. The colistin minimum inhibitory concentration (MIC) was ≥ 512 µg/mL, indicating high-level resistance. In contrast, DFU32 remained susceptible to piperacillin/tazobactam, aztreonam, meropenem, ciprofloxacin, and fosfomycin.

### Assembly and annotation features of DFU32 draft genome

The genome assembly of strain DFU32 consisted of 60 contigs with a total length of 5,196,771 bp and a GC content of 52.99%. The assembly had an N50 value of 462,353 bp and an L50 of 5. Genome completeness was estimated at 100%.

MOB-suite analysis of the draft genome did not classify any contig as a plasmid. However, four contigs contained plasmid-associated features. Contig 15 (58,689 bp) carried an IncR (incompatibility group R) replicon, whereas contig 18 (47,771 bp) carried an IncC (incompatibility group C) replicon together with an MPF_F (F-type mating pair formation) system and a MOBH (MOBH-family) oriT (origin of transfer) sequence. Contig 25 (21,255 bp) was associated with MPF_F (F-type mating pair formation) features, whereas contig 26 (11,732 bp) contained a replicon assigned to rep_cluster_1254.

The NCBI PGAP identified a total of 5,024 genes, including 4,932 coding DNA sequences (CDSs). The CDSs include 4,840 protein-coding genes and 92 RNA genes comprising various types of non-coding RNA essential for cellular functions.

### Population analysis of *C. koseri* genomes and phylogenetic placement of DFU32

Strain DFU32 was assigned to sequence type ST1172 using FastMLST. At the time of analysis, the NCBI genomic database contained 518 *C. koseri* genomes, including 36 complete genomes with a total of 48 distinct STs. Out of all strains with public genomes, 58 (11.2%) harboured novel alleles, and 28 (5.4%) displayed previously unreported allelic profiles (new ST). The most prevalent ST was ST854, accounting for 169 strains (32.6%), followed by ST64 (*n* = 64; 12.4%) and ST862 (6.2%). STs of all *C. koseri* strains are listed in Supplementary Table 1.

To further investigate the genomic relatedness of DFU32 and other *C. koseri* strains, genome-wide SNP analysis was performed, followed by assessment of the contribution of homologous recombination to the observed genetic variation. Gubbins identified a relatively low contribution of homologous recombination to the observed sequence variation, with an average of 177.5 ± 545.4 SNPs per comparison. Of these, 7.3 ± 41.1 SNPs (3.3 ± 15.3%) were located within predicted recombination regions, while 170.2 ± 541.6 SNPs occurred outside recombination regions. An average of 0.6 ± 3.0 recombination blocks were detected, encompassing 97.1 ± 545.3 recombinant bases. The mean (r/m) ratio was 0.2 ± 1.7, indicating that recombination generally contributed less to nucleotide variation than point mutation. A detailed summary of the Gubbins-predicted recombination and SNPs distribution is provided in Supplementary Table 2.

**Fig. 1:**
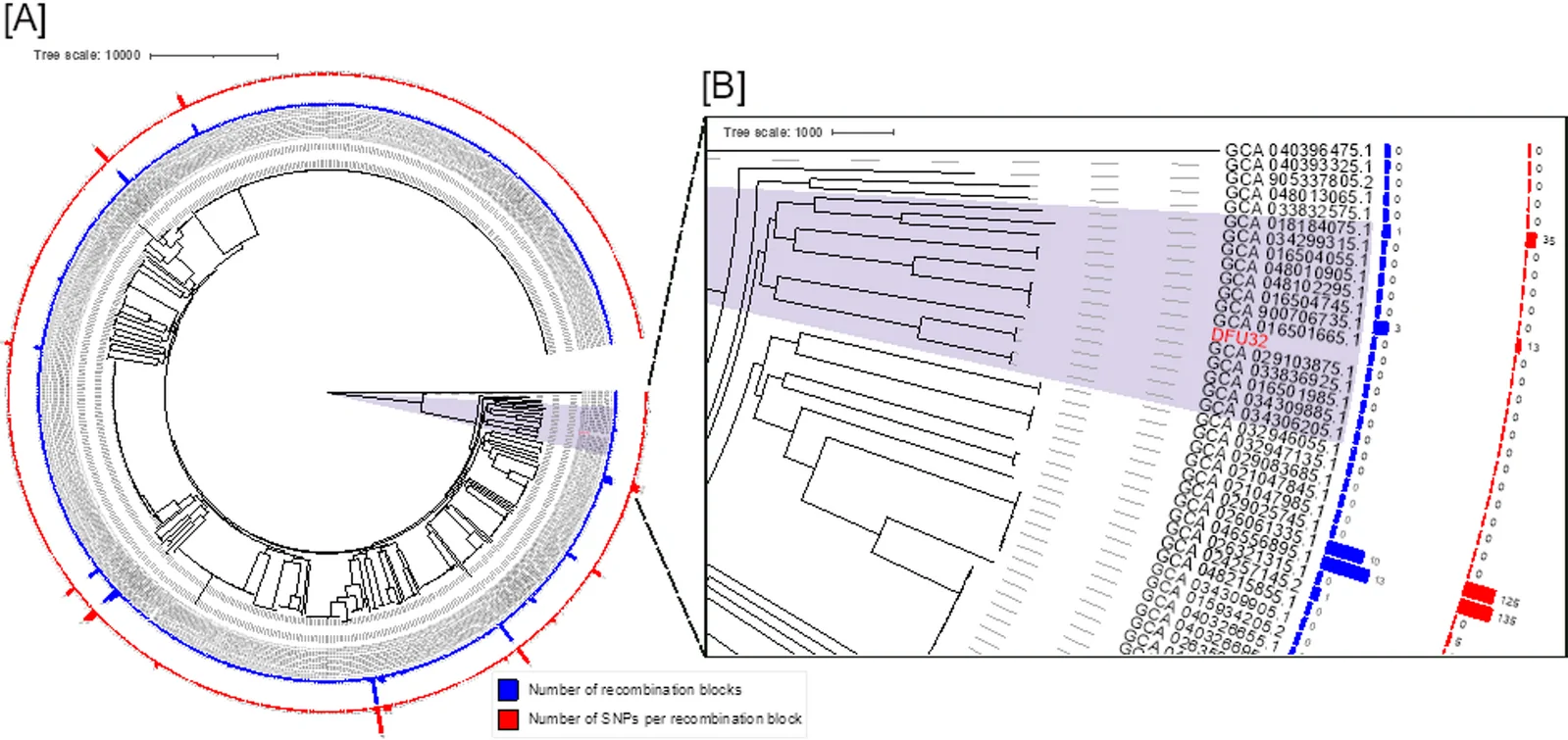
SNP-based phylogenetic analysis of *C. koseri*. **A** Core genome SNP–based phylogenetic tree showing the phylogenetic placement of isolate DFU32 among 518 *C. koseri* strains with publicly available genome sequences in the NCBI database. **B** Enlarged view of the phylogenetic cluster containing DFU32, highlighting its closest related strains based on core genome SNP analysis. The cluster containing DFU32 is highlighted in purple. Blue bars show the number of recombination blocks per isolate, and red bars show the number of SNPs per recombination block for each isolate

The SNP analyses of recombination-free alignment and phylogeny (Fig. 1) indicated that DFU32 clustered closely with strain CB00152 (GCA_016501665.1), isolated in the United States in 2019, differing by only 22 core genome SNPs; both isolates belonged to the same sequence type (ST1172). DFU32 was also related to strain NCTC10849 (GCA_900706735.1), collected in the USA in 2014, that have undefined sequence type, differing by 23 SNPs. We could not relate our isolate to other Egyptian or Arabian isolates due to lack of metadata. The full SNP distance matrix is provided in Supplementary Table 3**.**

### DFU32 Virulome

The combined analysis of the three VF detection tools, VFanalyzer, ABRicate, and VirulenceFinder, revealed a broad repertoire of predicted VGs in DFU32, encompassing 24 virulence factor (VF) classes. VFanalyzer identified the largest number of VGs (216 genes). Five genes were identified by all three tools, while 58 genes were shared between VFanalyzer and ABRicate. The most represented VF classes were iron uptake (48 genes) and adherence-associated factors (39 genes). Additional predicted virulence-associated functions included biofilm formation, efflux, chemotaxis and motility, toxins, invasion, among others. The complete distribution of virulence factors, including the corresponding genes, DFU32 locus tags, and the contribution of each of the three VG detection tools, is presented in Supplementary Table 4.

### DFU32 Resistome

Analysis of the DFU32 genome using AMRFinder revealed the presence of 21 AMR genes, potentially conferring resistance to at least ten different antimicrobial classes. The identified genes encode a variety of resistance mechanisms, including antibiotic inactivation, target modification or protection, efflux pumps, and target replacement. Among the detected AMR genes, those associated with resistance to aminoglycosides were the most abundant (7/21; 33.3%), followed by genes conferring resistance to beta-lactams (5/21; 23.8%). A detailed list of the detected resistance genes, their associated antimicrobial classes, and predicted mechanisms is provided in Table 1. Chromosomal mutations potentially associated with colistin resistance were investigated through comparative genomic analysis using *C. koseri* ATCC BAA-895 as the reference genome. No mutations were identified in the *pmrCAB* operon. Within the PhoP/PhoQ two-component regulatory system, no amino acid substitutions were detected in *phoP*, while a single substitution (V381I) was observed in phoQ. Additionally, no mutations were detected in the lipid A biosynthesis genes (*lpxA*, *lpxC*, and *lpxD*) or in the negative regulator gene *mgrB*. Mutation analysis of the *gyrA* and *parC* genes identified a single missense mutation in *gyrA*, resulting in a Y211H amino acid substitution, while no mutations were detected in *parC*.

**Table 1:** AMR genes detected in the DFU32 genome using AMRFinder

Antimicrobial Class	Resistance Gene	Resistance Mechanism
Beta-lactams	*bla*_CMY−2_	antibiotic inactivation
	*bla*_OXA−9_	antibiotic inactivation
	*bla*_CTX−M−15_	antibiotic inactivation
	*bla*_TEM_	antibiotic inactivation
	*bla*_CKO_	antibiotic inactivation
Aminoglycosides	*aph(6)-Id*	antibiotic inactivation
	*aph(3’’)-Ib*	antibiotic inactivation
	*armA*	antibiotic target alteration
	*aac(3)-IId*	antibiotic inactivation
	*aadA2*	antibiotic inactivation
	*aac(6’)-Ib*	antibiotic inactivation
	*aadA1*	antibiotic inactivation
Fosfomycin	*fosA7*	antibiotic inactivation
Macrolide	*mph(E)*	antibiotic inactivation
Macrolide/streptogramin	*msr(E)*	antibiotic target protection
Phenicol	*floR*	antibiotic efflux
Quinolone	*qnrS1*	antibiotic target protection
Sulfonamide	*sul2*	antibiotic target replacement
	*sul1*	antibiotic target replacement
Tetracycline	*tet(A)*	antibiotic efflux
Trimethoprim	*dfrA12*	antibiotic target replacement

**Fig. 2:**
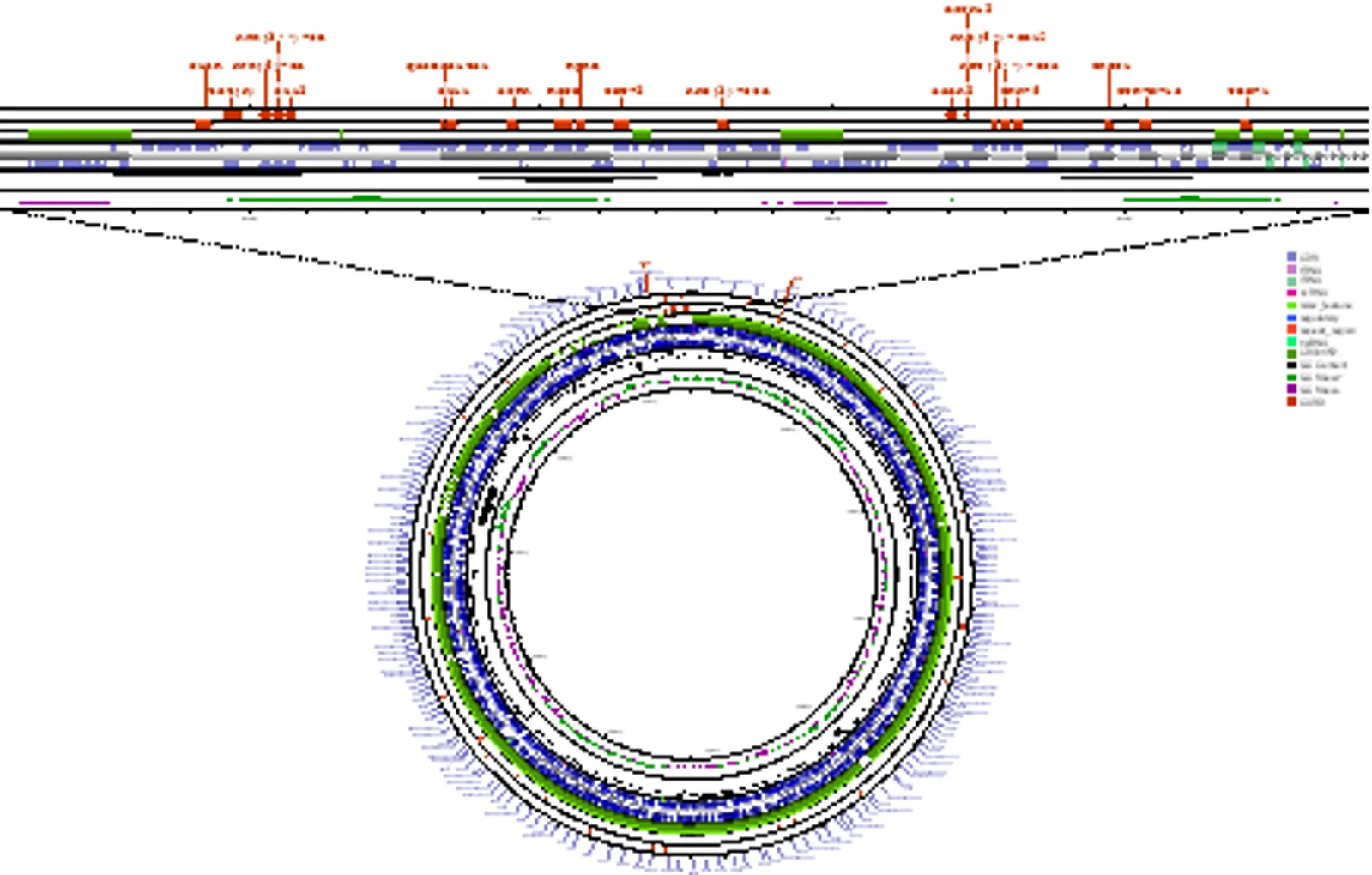
Pairwise genome alignment of DFU32 against the closely related strain CB00152 (ST1172). The comparison highlights AMR genes uniquely exist in DFU32, with co-localized gene clusters indicated along individual contigs

Pairwise sequence alignment of DFU32 with the closely related strain CB00152, which belongs to the same ST, indicated that most AMR genes were uniquely carried by DFU32 (Fig. 2). Although these genes were located on relatively small contigs, which limited precise characterization of the genetic elements carrying them, several lines of evidence supported their potential plasmid association. Multiple AMR genes were co-localized on the same contigs, including *floR*, *tet(A)*, *aph(6)-Id*, *aph(3’’)-Ib*, and *sul2* on contig 25; *qacEΔ1*, *sul1*, *armA*, *msr(E)*, and *mph(E)* on contig 26; *aac(6’)-Ib10*, *ant(3’’)-IIa*, and *bla*_OXA−9_ on contig 33; and *aadA2* and *dfrA12* on contig 32. All four contigs showed 100% coverage and ≥ 99% identity to plasmid sequences from other Gram-negative bacterial species. In addition, MOB-suite analysis identified plasmid-associated features in contigs 25 and 26, including an MPF_F feature and a replicon assigned to rep_cluster_1254, respectively. The genes *aadA2* and *dfrA12* are included within a class I integron. Other genes were carried individually on small contigs, including *bla*_CMY−2_, *aac(3)-IId*, *qnrS1*, *bla*_CTX−M−15_, and *bla*_TEM−1_ genes.

The fosfomycin resistance glutathione transferase–encoding gene *fosA7* was located within a 57,414 bp integrative and conjugative element (ICE) that harboured genes encoding a type IV secretion system. The element was integrated at the chromosomal attachment site CGAAGGCCGGACTCG and was positioned adjacent to a tRNA-Leu (CAA) gene. Based on mobility-associated gene content, the element was classified as having a MOBH-type relaxase and a type G mating pair formation system.

### Resistome analysis of *C. koseri* public genomes

#### Prevalence of resistance genes

A total of 140 resistance genes, including gene variants, were identified among *C. koseri* genomes, conferring resistance to at least 14 classes of antimicrobial agents. Most genomes harboured at least one MAL/CKO family class A β-lactamase–encoding gene. Based on the distribution of these genes, the population was divided into two major lineages. The first lineage comprised genomes carrying *bla*_CKO_ (*n* = 247; 47.6%), while the second lineage included genomes carrying *bla*_MAL_ genes (*n* = 266; 51.3%), subdivided into *bla*_MAL−1_ (*n* = 180; 67.7%) and *bla*_MAL−2_ (*n* = 86; 32.3%). Only three strains lacked all three genes - *bla*_CKO_, *bla*_MAL−1_, and *bla*_MAL−2_: GCA_049190535.2 (novel ST), GCA_900461075.1 (ST1154), and GCA_900446925.1 (novel ST). In contrast, three strains (GCA_048215345.1, GCA_017714815.1, and GCA_045746615.1) carried both *bla*_CKO_ and *bla*_MAL−1_; all belonged to ST854.

**Fig. 3:**
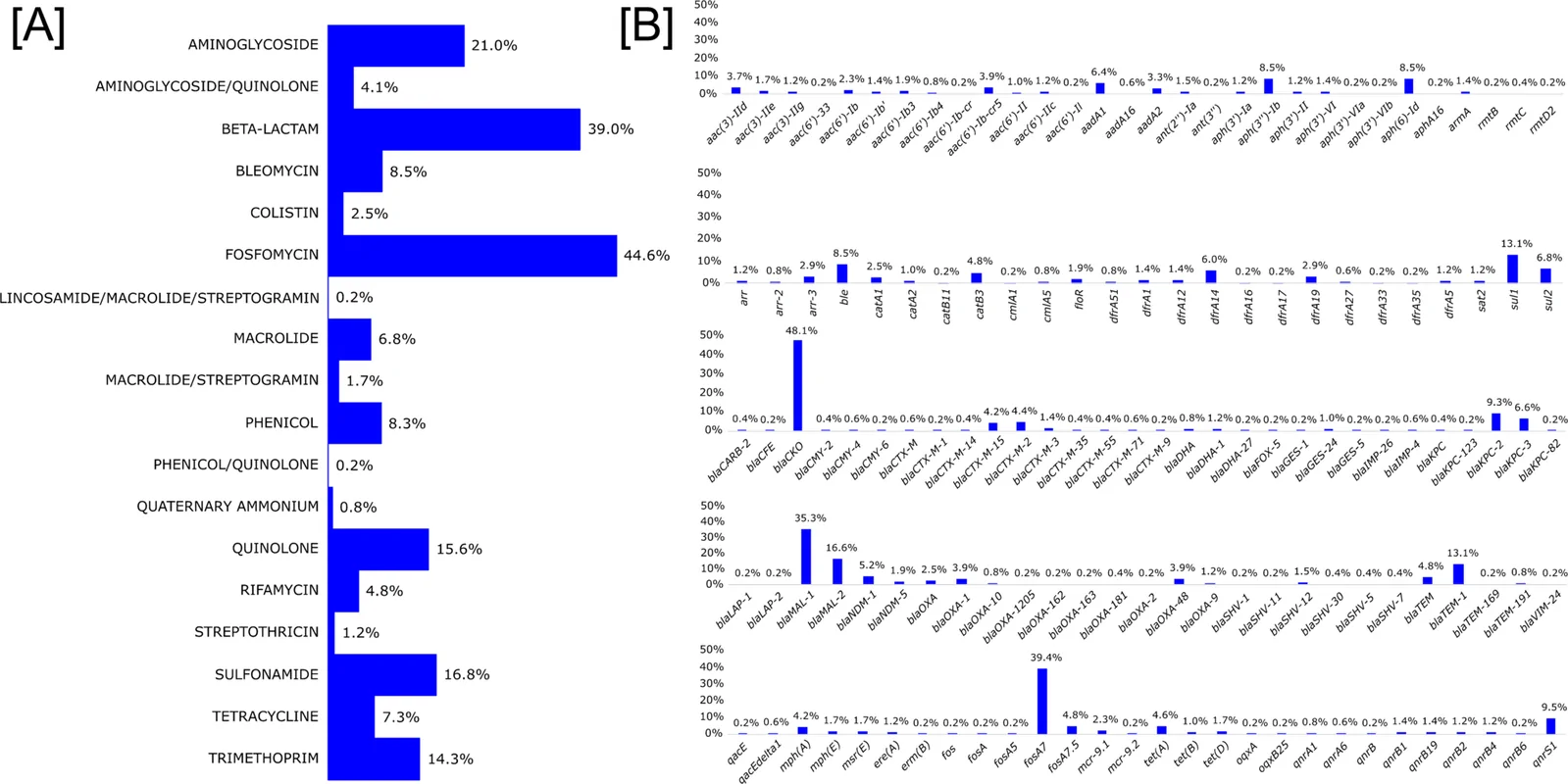
Distribution of antimicrobial resistance genes among *C. koseri* genomes. **A** Bar chart showing the prevalence of resistance genes conferring resistance to different classes of antimicrobial agents across *C. koseri* genomes. **B** Prevalence of individual AMR genes detected among *C. koseri* genomes

At least 381 (73.6%) genomes carried acquired resistance genes. Following *bla*_CKO/MAL_, the second most prevalent AMR genes were *fos* gene variants that were carried by 233 genomes (44.8%). A total of thirteen carbapenemase-encoding genes were found, including *bla*_NDM−1_, *bla*_NDM−5_, *bla*_KPC_, *bla*_KPC−123_, *bla*_KPC−2_, *bla*_KPC−3_, *bla*_KPC−82_, *bla*_IMP−26_, *bla*_IMP−4_, *bla*_GES−1_, *bla*_GES−24_, *bla*_GES−5_, and *bla*_OXA−48_. Additionally, two variants of the *mcr* gene, *mcr-9.1* and *mcr-9.2*, were detected in *C. koseri* genomes. Collectively, 151 (29.1%) strains carried carbapenemase-coding genes, and 13 (2.5%) carried *mcr* genes. Figure 3 illustrates the prevalence of resistance genes to different classes of antimicrobial agents.

**Fig. 4:**
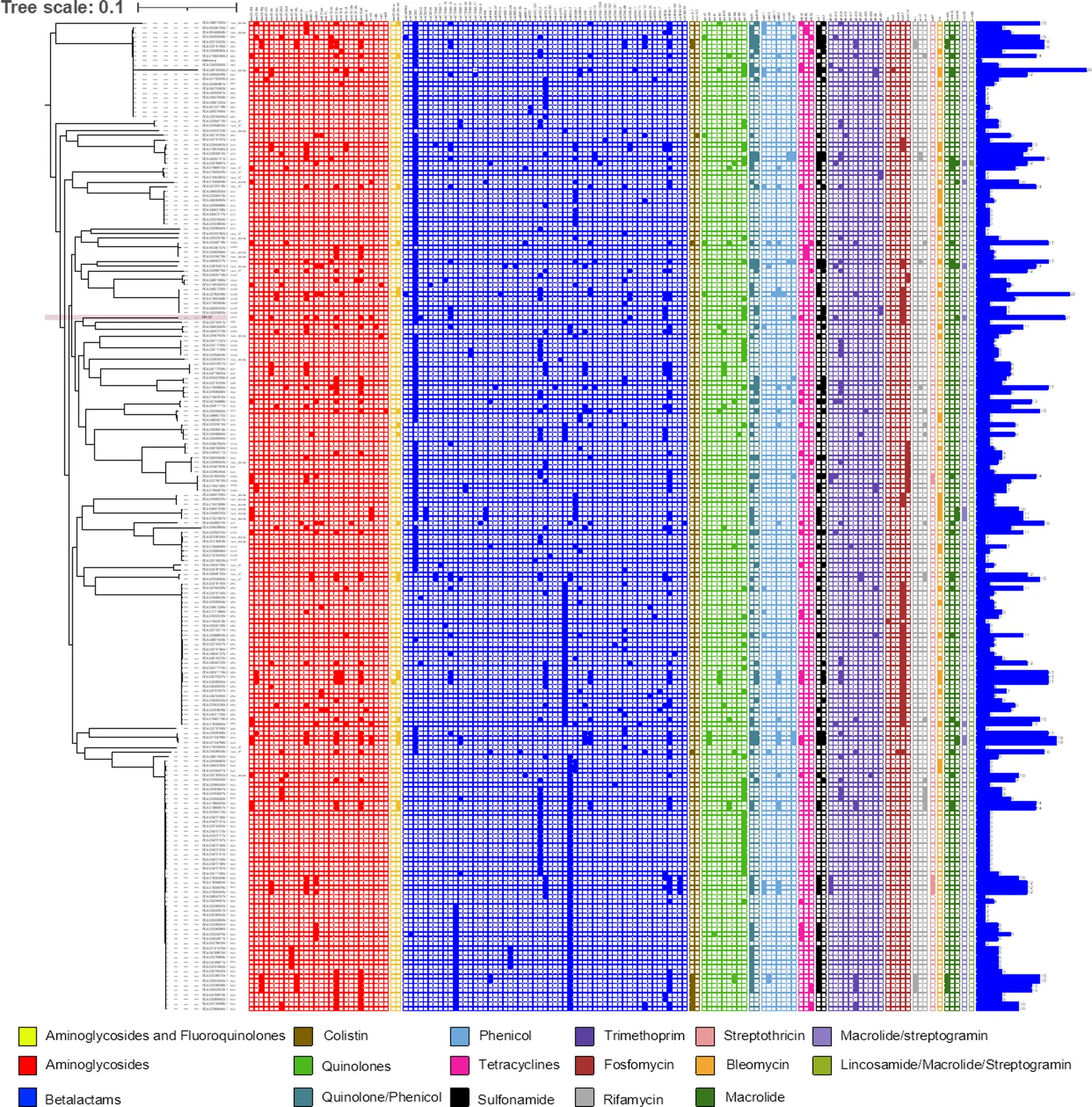
SNP-based phylogenetic tree of DFU32 and 381 *C. koseri* strains carrying acquired resistance genes. Isolates carrying only *bla*_CKO_ or *bla*_MAL_ together with *fosA7/fosA7.5* were removed from the tree for clarity. The tree is annotated with acquired resistance genes: solid squares indicate gene presence, and hollow squares indicate gene absence. The horizontal bars adjacent to each isolate represent the total number of resistance genes carried

Up to 26 AMR genes co-occurred within individual *C. koseri* strains (Fig. 4). Genomes of the MAL lineage carried a significantly higher number of co-existing AMR genes than those of the CKO lineage (Mann–Whitney U test, mean rank: MAL = 273.88, CKO = 238.83, *p* = 0.005). Within the MAL lineage, MAL-2 genomes also carried more AMR genes than MAL-1 genomes (Mann–Whitney U test, mean rank: MAL-2 = 152.45, MAL-1 = 124.44, *p* = 0.002).

The most frequent AMR gene combinations were *bla*_MAL−1_ and *fosA7* (139 strains; 26.8%), *bla*_CKO_ and *fosA7* (17 strains; 3.3%), *bla*_MAL−2_, *bla*_KPC−2_, and *qnrS1* (13 strains; 2.5%), *bla*_CKO_ and *fosA7.5* (12 strains; 2.3%), *bla*_CKO_ and *bla*_KPC−3_ (6 strains; 1.2%), *bla*_CKO_, *bla*_NDM−1_, and *ble* (6 strains; 1.2%), *bla*_MAL−2_, *aac(6’)-II*, *bla*_CTX−M−2_, *bla*_GES−24_, and *sul1* (5 strains; 1.0%), and *bla*_MAL−2_ and *bla*_CTX−M−2_ (5 strains; 1.0%). Combinations of carbapenemase and *mcr* genes were detected in only four strains (0.8%). A comprehensive summary of the AMR gene profiles identified across the analysed *C. koseri* genomes is provided in Supplementary Table 1.

#### Distribution of resistance genes in different STs

**Fig. 5:**
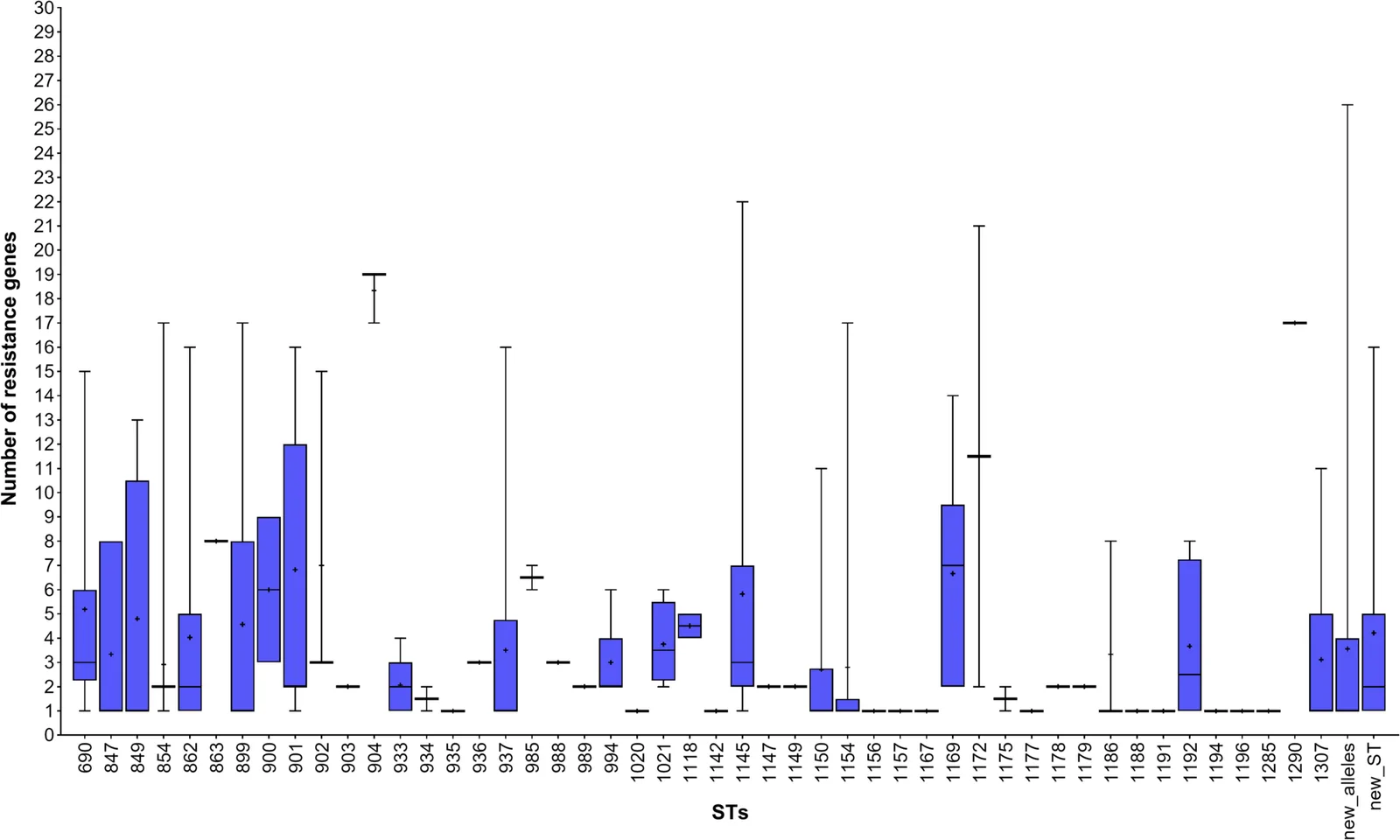
Box-and-whisker plot showing the total number of resistance genes carried by different sequence types of *C. koseri*. Each box represents the interquartile range, the horizontal line within the box indicates the median, the whiskers show the minimum and maximum values, and the plus sign (+) within each box represents the mean number of resistance genes

Among the STs represented by more than five genomes, the highest resistance gene burdens were observed in ST901 (median = 2.00; mean = 6.82), ST1169 (median = 7.00; mean = 6.67), ST1145 (median = 3.00; mean = 5.82), and ST690 (median = 3.00; mean = 5.19). The distribution of resistance genes across all STs is shown in Fig. 5. For STs represented by five genomes or fewer, the greatest resistance gene burdens were observed in ST904 (median = 19.00; mean = 18.33), ST1290 (median = 17.00; mean = 17.00), and ST1172 (median = 11.50; mean = 11.50). However, because these STs are represented by limited numbers of genomes, the true resistance gene burden within these lineages cannot be reliably estimated.

Colistin resistance–associated *mcr* genes were detected in nine STs, with ST690 being the predominant lineage, harbouring four *mcr*-positive strains, followed by ST862, which included two positive strains. On the other hand, carbapenemase-encoding genes were detected in nearly half of the *C. koseri* STs (21 out of 48 STs; 43.8%), in addition to several novel STs. ST690 was the predominant lineage, carrying the highest total number of carbapenemase genes (*n* = 37), primarily *bla*_KPC_ (*n* = 24), followed by *bla*_NDM_ (*n* = 7) and *bla*_OXA−48_ (*n* = 3), with sporadic detection of *bla*_GES_ (*n* = 3). ST854 was the second most prominent lineage (*n* = 30), displaying a more heterogeneous carbapenemase profile, including *bla*_KPC_ (*n* = 13), *bl*a_OXA−48_ (*n* = 9), *bla*_NDM_ (*n* = 5), and *bla*_IMP_ (*n* = 3).

**Fig. 6:**
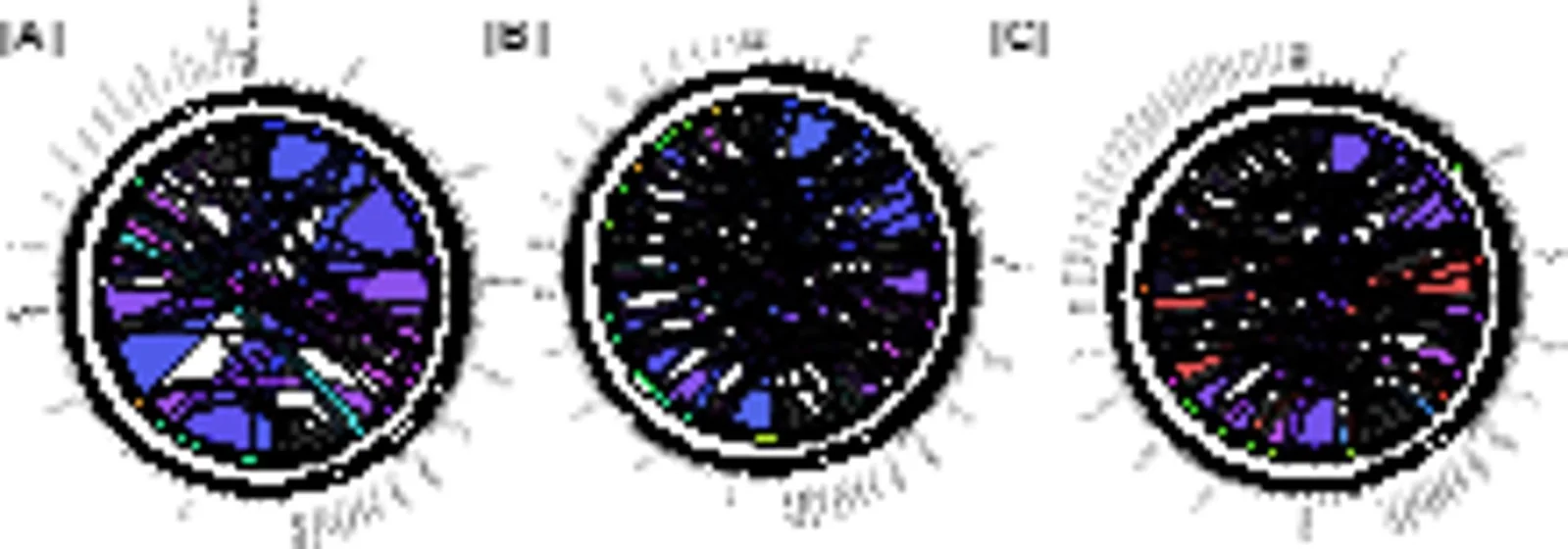
Circos plots of the global distribution, year of isolation, and sequence types of *C. koseri* strains carrying carbapenemase- and *mcr*-coding genes. **A** The global distribution of carbapenemase- and *mcr*-coding genes by country of isolation. **B** The distribution of carbapenemase- and *mcr*-coding genes according to the year of isolation. **C** Circos plot depicting the association between carbapenemase- and *mcr*-coding genes STs. In all panels, ribbon widths are proportional to the number of reported isolates. Figures were generated using Circos Table Viewer v0.63-10 [42]

Strains with novel STs collectively harboured 25 carbapenemase genes, predominantly *bla*_KPC_ (*n* = 14) and *bla*_NDM_ (*n* = 8), with less frequent occurrence of *bla*_OXA−48_ (*n* = 2) and *bla*_GES_ (*n* = 1). Several additional STs, including ST862 and ST933, carried moderate numbers of carbapenemase genes (7–9 per ST), typically dominated by *bla*_KPC_ and *bla*_NDM_ variants. The global distribution, year of isolation, and STs of *C. koseri* strains carrying carbapenemase- and *mcr*-coding genes are presented in Fig. 6. Distribution of carbapenemase-coding genes in different STs is provided in Supplementary Tables 5, and the detailed information on *C. koseri* strains carrying carbapenemase- and *mcr*-coding genes is provided in Supplementary Table 6**.**

#### Context of carbapenem and colistin resistance genes

**Fig. 7:**
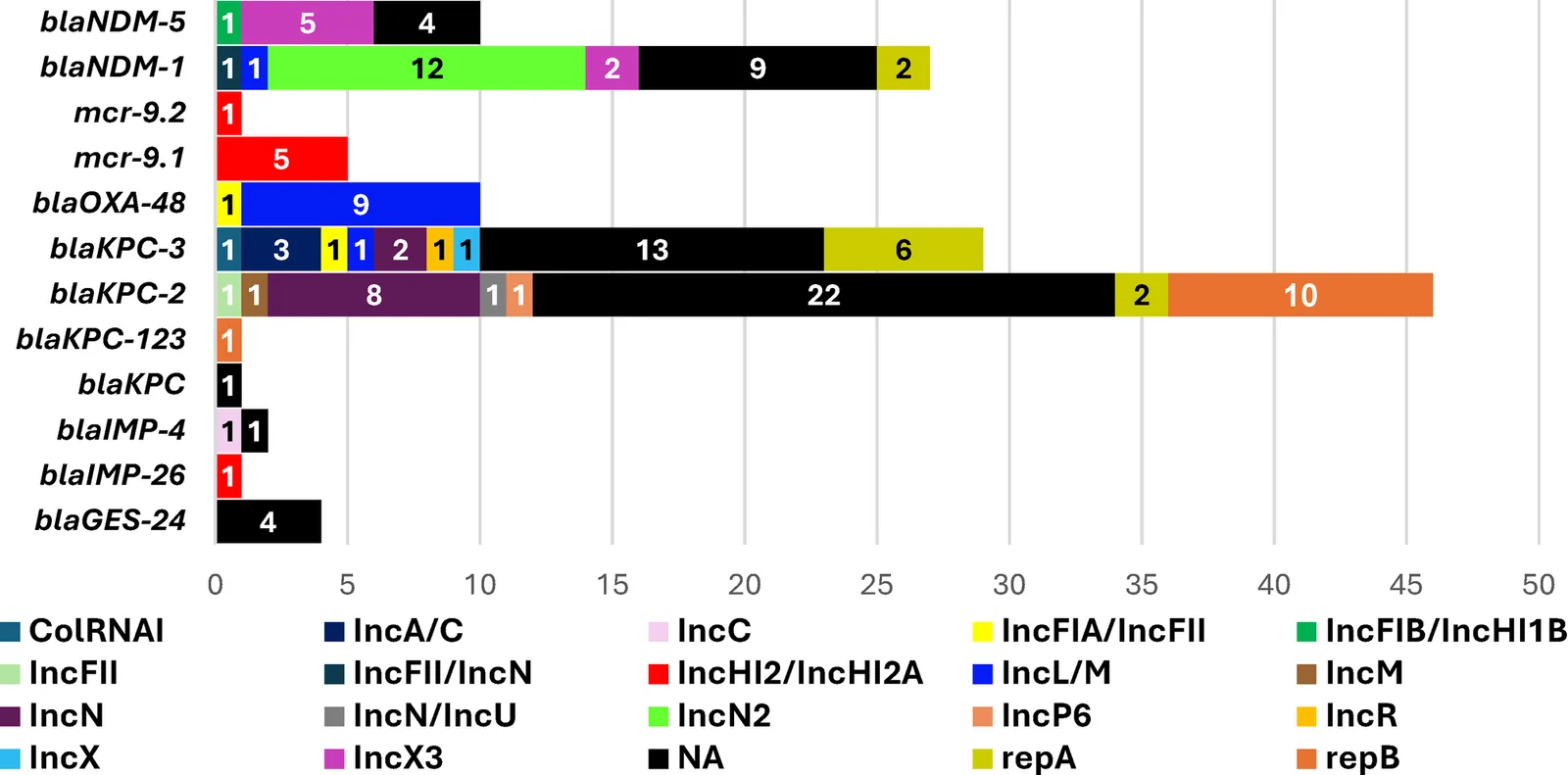
Stacked bar chart showing the prevalence of different replicon types across individual gene types. Each bar segment represents the number of isolates carrying the specified gene-replicon combination

Carbapenemase- and *mcr*-encoding genes were predominantly located on contigs matching plasmid sequences (*n* = 137; 82%). A smaller proportion were identified on short contigs aligning to both chromosomal and plasmid sequences (*n* = 25; 15%). Plasmid replicon types could be assigned to 83/137 (60.6%) of the plasmid-associated contigs. The most prevalent replicon types were IncN2 (*n* = 12; 8.8%), followed by IncL/M and repB (each *n* = 11; 8.0%), and repA and IncN (each *n* = 10; 7.3%). All plasmid replicon types associated with carbapenemase and mcr genes are detailed in Fig. 7.

**Fig. 8:**
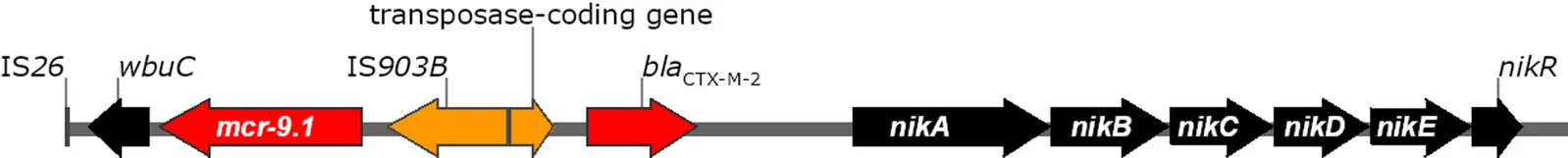
Genetic map of the *mcr-9.1*–carrying transposon integrated into the chromosome of *C. koseri* strain IR025 (GenBank accession no. NZ_JAXBAE010000013.1), showing the flanking IS*26* and IS*903B* family transposase-coding genes

The *mcr-9.1* gene was identified on contigs corresponding to chromosomal sequences in two isolates from Japan belonging to sequence type ST690: strain TUM3761 (collected in 2007) and strain IR025 (collected in 2019). In both isolates, *mcr-9.1* was embedded within a transposon flanked by an IS*26* and an IS*903B* family transposases. This transposon was positioned immediately upstream of a *bla*_CTX−M−2_ gene (Fig. 8). Sequence analysis demonstrated that the transposon shared 100% identity with plasmid pXXB1403 from *Salmonella enterica* subsp. *diarizonae* serovar b strain XXB1403 (GenBank accession CP059887.1). It also exhibited 99.92% sequence identity with related plasmids identified in *Citrobacter braakii*, *Escherichia coli*, *Klebsiella pneumoniae*, and *Enterobacter hormaechei*. Notably, chromosomal integration of this transposon carrying *mcr-9.1* appeared unique to strains IR025 and TUM3761, with no comparable chromosomal insertions identified in the NCBI database.

The *bla*_KPC−2_ gene was located on the chromosome of two strains isolated from patients in China in 2023 (K2521 and K2721), both representing a novel ST. Notably, the gene was embedded within an IS*26*-bracketed transposon present in two copies in each strain. These two copies shared 99.98% nucleotide identity. The transposon exhibited 100% coverage and 100% identity to a region of the pKPC-DD02201 of *Klebsiella pneumoniae* subsp. *pneumoniae* strain DD02201 (GenBank accession: P096241.1), although with a different genetic organisation. One of the two *bla*_KPC−2_–harbouring transposon copies was located adjacent to IS*Ecp1*, which was associated with two β-lactamase genes, namely *bla*_TEM−1_ (interrupted by IS*26*) and *bla*_CTX−M−3_.

## Discussion

DFUs are complex polymicrobial environments that harbour diverse microorganisms, including previously unrecognised bacterial species. The recent identification of *Providencia pseudovermicola* sp. nov. from infected DFUs in Egypt highlights this diversity and the value of WGS for resolving novel or misidentified pathogens [43]. Additionally, our recent WGS-based study of MDR *Pseudomonas aeruginosa* isolated from DFUs further highlights the genomic diversity and adaptive potential of DFU-associated pathogens, reinforcing the value of genomic surveillance in understanding their clinical impact [44]. DFUs may also contain opportunistic Gram-negative bacteria that remain poorly studied at both genomic and epidemiological levels. Among these, *C. koseri* has received little attention in Egypt despite its possible clinical importance. The present study describes the first draft genome of *C. koseri* isolated from a DFU patient in Egypt. The isolate exhibited an MDR phenotype and was notably non-susceptible to last-resort therapeutic agents, including colistin and tigecycline, highlighting the potential clinical threat posed by this emerging pathogen.

Strain DFU32 was assigned to sequence type ST1172 and clustered with *C. koseri* strains originating from the United States. This suggests that this lineage is not geographically restricted and may be part of a widely disseminated clonal background. MLST analysis of publicly available *C. koseri* genomes further revealed substantial allelic diversity, with 58 strains carrying novel alleles and 28 strains exhibiting previously unreported allelic profiles. These findings highlight gaps in the current MLST database. They also underscore the need for systematic curation and updating of *C. koseri* entries in the PubMLST repository. Such efforts would improve sequence-typing accuracy and strengthen epidemiological surveillance of this species.

Genome-wide in silico analysis of DFU32 identified several predicted VF determinants with potential relevance to colonisation and persistence in chronic diabetic foot wounds. Notably, genes encoding type I and III fimbriae, type IV pili, *E. coli* common pilus (ECP), and curli fibers were identified. These structures may promote adhesion to host tissues [45–47] and contribute to biofilm development, which is an important feature of chronic wound persistence [48]. DFU32 also carried multiple iron-acquisition systems, including aerobactin, enterobactin, and haem-uptake determinants. It also harboured the high-pathogenicity island (HPI), first described in highly pathogenic *Yersinia* strains. Yuan et al., [49] subsequently reported the HPI as unique to *C. koseri* among *Citrobacter* species and demonstrated its essential role in *C. koseri* pathogenicity. Collectively, these systems may enhance bacterial fitness under the iron-limited conditions imposed by host nutritional immunity [50]. DFU32 carried an extensive repertoire of genes associated with flagellar assembly, motor function, and chemotaxis. These genes may support bacterial motility and chemotactic navigation, potentially facilitating wound-surface colonisation and biofilm establishment [51]. Notably, *Citrobacter* is not currently supported as a reference genus or species in commonly used virulence-factor detection tools. Therefore, our findings support the use of a multi-tool, homology-based approach for a more comprehensive assessment of the virulence potential of *Citrobacter* isolates.

Genomic analysis of DFU32 revealed a substantial β-lactam resistance burden, with at least five β-lactamase–encoding genes identified. In addition to the intrinsic chromosomal class A β-lactamase gene *bla*_CKO_ [52] characteristic of *C. koseri*, the isolate harboured multiple acquired β-lactamase genes. These included narrow-spectrum enzymes (*bla*_TEM_ and *bla*_OXA−9_) as well as extended-spectrum β-lactamases and cephalosporinases (*bla*_CTX−M−15_ and *bla*_CMY−2_). The coexistence of intrinsic and acquired β-lactamase determinants likely contributes to the broad β-lactam resistance phenotype observed in DFU32. The emergence of novel β-lactamase variants has previously been documented in *C. koseri*, including *bla*_TEM−134_ [53], *bla*_CTX−M−2_ [54], and *bla*_CTX−M−35_ [55]. These reports illustrate the remarkable genetic plasticity of this species. Aminoglycosides were once considered part of first-line therapy for *Citrobacter* infections; however, DFU32 harboured at least seven aminoglycoside resistance genes. These included the broad-spectrum resistance determinant *armA*, encoding a 16 S rRNA (guanine(1405)-N(7)-methyltransferase, which is known to confer high-level resistance to nearly all clinically relevant aminoglycosides [56]. Additional resistance determinants targeting multiple antimicrobial classes were also identified, consistent with the observed phenotypic resistance profile.

Although DFU32 carries *fosA7*, which encodes a fosfomycin resistance glutathione transferase, the isolate remained phenotypically susceptible to fosfomycin. This finding supports renewed interest in repurposing older antibiotics [57, 58]. While direct functional evidence for *fosA7* activity in *C. koseri* remains limited, recent work has described *fosA7* as a potentially silent fosfomycin resistance gene in *Salmonella*, where it has been detected in fosfomycin-susceptible isolates. Nevertheless, its mobilization onto high-copy plasmids or other mobile genetic elements could facilitate future emergence of resistance [59]. Similarly, although *qnrS1* was detected, DFU32 remained susceptible to ciprofloxacin. As reported by Jacoby et al., [60], plasmid-mediated quinolone resistance (PMQR) determinants generally confer only low-level reductions in quinolone susceptibility and may not reach clinical resistance breakpoints in the absence of additional mutations within the quinolone resistance–determining region (QRDRs). The detected *gyrA* substitution (Y211H) in DFU32 lies outside the QRDR and is therefore unlikely to confer fluoroquinolone resistance [61, 62].

Colistin resistance in DFU32 was associated with a missense substitution in the *phoQ* gene (V381I), which is part of the PhoP/Q two-component system. It plays a key role in colistin resistance in *Enterobacteriaceae* by regulating lipid A modifications that reduce antibiotic binding [63]. Because polymorphisms in colistin resistance-associated loci may be ST-specific, comparisons with unrelated reference genomes can lead to misinterpretation [64]. The V381I substitution was also identified in the only other available ST1172 genome, strain CB00152, for which the colistin susceptibility profile is unknown. Furthermore, no *mcr* genes or other known chromosomal colistin-resistance-associated mutations were identified in DFU32. Therefore, although the *phoQ* V381I substitution represents a potential genetic correlate of the observed colistin resistance, its causal role cannot be definitively established based on the available data. Functional validation will be required to determine whether this substitution contributes to colistin resistance or whether additional, currently uncharacterised mechanisms are involved.

Although the genetic context of most resistance genes in DFU32 could not be fully resolved, comparative genome analysis with CB00152 (Fig. 2) revealed that DFU32 uniquely harboured at least 20 resistance genes, indicating likely acquisition via horizontal gene transfer.

The present genomic analysis also provides a comprehensive overview of the AMR landscape in *C. koseri*. At least 140 acquired and intrinsic AMR genes conferring resistance to at least 14 antimicrobial classes were identified in *C. koseri* genomes. A high proportion of genomes carried acquired resistance determinants (73.6%). The frequent co-occurrence of multiple AMR genes, with up to 26 genes in a single genome, illustrates the MDR potential of *C. koseri*. Although combinations of carbapenemase and *mcr* genes were rare, their presence in individual strains signals the emergence of extensively drug-resistant phenotypes that may limit therapeutic options.

Analysis of the AMR in the context of STs showed that the highest resistance gene burdens were observed in ST904, ST1290, and ST1172. These lineages were represented by only a few genomes, limiting the reliability of burden estimates and their epidemiological interpretation. In contrast, among STs represented by larger numbers of genomes, ST901, ST1169, ST1145, and ST690 exhibited the greatest resistance gene loads, suggesting stable accumulation of resistance determinants within these circulating lineages. ST690 emerged as one of the most epidemiologically significant lineages. This ST ranked among the top STs in terms of resistance gene burden and also showed the widest distribution of critical resistance determinants, such as carbapenemase genes and *mcr* genes.

Consistent with the findings of Osei Sekyere and Reta [65], who analysed 82 *C. koseri* genomes, the species appears to be structured into two principal lineages defined by chromosomal class A β-lactamase genes. All except three genomes harbour either *bla*_CKO_ or *bla*_MAL_ variants, supporting their utility as stable phylogenetic markers. Notably, genomes belonging to the MAL lineage, particularly MAL-2, harboured significantly higher numbers of co-occurring AMR genes, indicating higher potential for acquiring and maintaining resistance determinants. Whether this translates into higher phenotypic resistance in this lineage remains to be investigated. Fosfomycin resistance genes were the second most prevalent determinants, detected in nearly half of the genomes. Despite their high prevalence, *fosA* gene variants may not always translate into phenotypic resistance [59].

Among the AMR genes identified in this species (Figs. 3 and 4), the widespread distribution of carbapenemase genes is especially alarming. Carbapenemase determinants were present in 29.1% of genomes and distributed across nearly half of the identified STs, highlighting their successful horizontal gene transfer across multiple STs. The predominance of *bla*_KPC_ variants, followed by *bla*_NDM_ and *bla*_OXA−48_, mirrors global trends in carbapenem resistance among *Enterobacterales* and indicates ongoing interspecies gene exchange [66]. Furthermore, the detection of *mcr-9* variants in multiple lineages, is alarming given their association with MGEs and the role of colistin as a last-resort therapeutic option.

With respect to geographic distribution, *bla*_KPC−2_ was predominantly identified in strains from China, whereas *bla*_KPC−3_ was most frequently detected in isolates from the United States. Most *bla*_NDM−1_–positive strains originated from Singapore, while *bla*_NDM−5_ was mainly reported from the United States. In contrast, *bla*_OXA−48_, *bla*_GES−24_, and *mcr-9.1* were largely associated with isolates from Japan, and *bla*_IMP−4_ was primarily detected in strains from Australia. However, these patterns may not accurately reflect the true global distribution of carbapenemase and *mcr* genes in *C. koseri*. Instead, they likely mirror the availability of WGS data from specific countries. Limited sequencing capacity and underreporting in other regions may result in an underestimation of the actual global burden and dissemination of these resistance determinants. Temporal analysis revealed shifting patterns in the emergence of carbapenemase and colistin resistance genes. *bla*_IMP_ variants were the predominant carbapenemase genes prior to 2004. Beginning in 2012, *bla*_KPC_ and *bla*_OXA−48_ started to appear in *C. koseri* genomes, followed by the emergence of *bla*_NDM−1_ in 2014. Notably, *mcr-9.1* was identified in genomes from isolates collected as early as 2007, whereas *mcr-9.2* was detected more recently, first appearing in 2022.

Plasmids played a dominant role in the dissemination of carbapenemase and *mcr* genes, with more than 80% located on plasmid-associated contigs. The diversity of replicon types, particularly IncN2 and IncL/M, highlights the involvement of broad host-range plasmids in resistance gene spread. The chromosomal integration of *mcr-9.1* in ST690 isolates represents a notable finding of our analysis. The association of this gene with IS26 and IS903B and its proximity to *bla*_CTX−M−2_ suggest a composite mobile element capable of co-mobilizing other resistance determinants. A similar genetic background has been previously reported in *Enterobacter hormaechei* from Lebanon [67]. Most *mcr* genes are associated with conjugative plasmids that facilitate horizontal transfer. Exceptions include chromosomal *mcr-6* in *Moraxella* sp., and *mcr-9* reported on plasmids or integrated into the chromosome in *Escherichia coli*, *Salmonella enterica*, and *Enterobacter hormaechei* [67]. The current study reports *C. koseri* as another Gram-negative species that harbours a chromosomally associated *mcr* gene, further highlighting the potential for stable maintenance and vertical transmission of colistin resistance beyond plasmid-mediated spread.

**Fig. 9:**
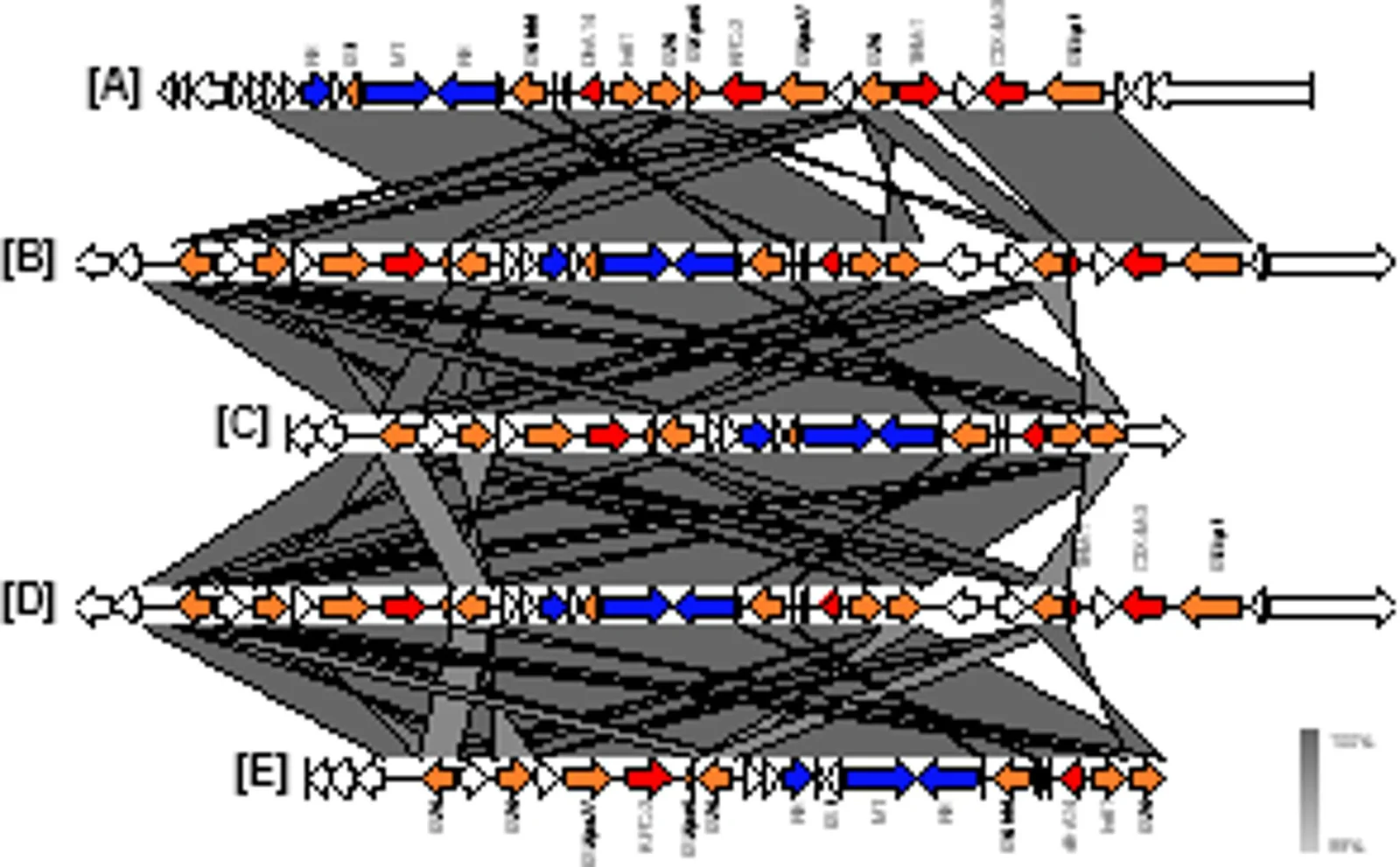
Gene maps showing comparison of the *bla*_KPC-2_–harbouring transposon. Panel **A** shows *Klebsiella pneumoniae* strain DD02201 plasmid pKPC-DD02201 (CP096241.1; 24,048–47,079). Panels **B** and **C** show *C. koseri* strain K2521 (NZ_CP136828.1; 310,580–337,045 and 483,924–501,865), while Panels **D** and **E** show *C. koseri* strain K2721 (NZ_CP136832.1; 4,727,335–4,753,800 and 4,900,225–4,917,449). Grey blocks indicate shared sequence regions. Arrows represent open reading frames (ORFs): orange for transposase or integrase genes, red for resistance genes, and blue for restriction–modification system genes

Similarly, the presence of chromosomal copies of *bla*_KPC−2_ embedded within an IS*26*-bracketed transposon (IS*26*–*recA*–IS*Kpn27*–*bla*_KPC−2_–IS*Kpn6*–IS*26*) in novel STs (Fig. 9) underscores the genomic plasticity of *C. koseri* and suggests potential stabilisation of carbapenem resistance independent of plasmid carriage. Immediately downstream of this element, an IS*Ecp1*-associated region carrying *bla*_CTX−M−3_ and *bla*_TEM−1_ was identified. The proximity of these elements raises the possibility of co-mobilization of the entire resistance unit, particularly given previous reports of IS*Ecp1*-mediated transposition of β-lactamase genes among *Enterobacteriaceae* [68–70].

The close similarity of this transposon to plasmid sequences described in *Klebsiella pneumoniae* underscores the role of interspecies genetic exchange in the evolution of resistance within *Enterobacterales*. The association of *bla*_KPC−2_ with IS*26*-bracketed transposons on plasmids has been widely documented in *K. pneumoniae* [71–74]. In contrast, chromosomal integration of *bla*_KPC−2_ has been reported only rarely in *Pseudomonas aeruginosa* [75], *Raoultella* spp. [76], *Acinetobacter baumannii* [77], and in *K. pneumoniae* [78], highlighting the unusual nature of such insertion events.

While our study provides a comprehensive genomic and resistome analysis of an understudied pathogen, it comes with some limitations. Due to financial constraints, long-read sequencing could not be performed for DFU32, and the publicly available genomes included in the analysis were likewise predominantly generated using short-read sequencing technologies. Consequently, the structure and genomic location of mobile genetic elements, particularly plasmids, could not be resolved with certainty. Future studies employing hybrid short- and long-read sequencing approaches will be necessary to accurately characterize these elements. Second, although DFU32 exhibited high-level colistin resistance, the underlying resistance mechanism could not be conclusively determined. Further investigations are planned to elucidate the genetic basis of this phenotype. Finally, the interpretation of geographic dissemination patterns and transmission dynamics was limited by the incomplete epidemiological metadata available for many publicly deposited genomes.

## Conclusion

This study provides the first genome-based characterization of an MDR *C. koseri* isolate (DFU32) from Egypt and the first large-scale analysis of the species-wide resistome using 518 publicly available genomes. DFU32 exhibited resistance to multiple antimicrobial classes, including last-resort agents such as colistin and tigecycline. It carried diverse resistance determinants, including ESBLs, aminoglycoside resistance genes, mobile genetic elements, and an integrative conjugative element harbouring *fosA7*. Comparative analysis of *C. koseri* genomes revealed two major chromosomal β-lactamase-defined lineages, with the MAL lineage, particularly MAL-2, showing a higher resistance gene burden and substantial diversity in resistome profiles. Plasmid-mediated carbapenemases were widely distributed in *C. koseri*, whereas mobilized colistin-resistance determinants were less frequent. The chromosomal integration events involving *mcr-9.1* and *bla*_KPC−2_, underscore the genomic plasticity of *C. koseri* and the potential for stable, plasmid-independent maintenance of critical resistance traits. ST690 emerged as a potential high-risk lineage combining MDR, carbapenemase, and colistin resistance determinants. These findings highlight the genomic plasticity and evolving resistance potential of *C. koseri* and support continued genomic surveillance, resistome monitoring, and antimicrobial stewardship.

## Supplementary Information

Below is the link to the electronic supplementary material.

Supplementary Material 1

Supplementary Material 2

## Data Availability

The genome sequence of DFU32 was submitted to the NCBI database under the Bioproject PRJNA1181229. Metadata of the genomes analyzed in this study are available in Supplementary Table 1.

## Abbreviations

AMR: Antimicrobial resistance.
ANI: Average nucleotide identity.
ATCC: American Type Culture Collection.
BLAST: Basic Local Alignment Search Tool.
bp: Base pairs.
CARB: Carbapenemase resistance (general usage in context).
CDS: Coding DNA sequence.
CLSI: Clinical and Laboratory Standards Institute.
dDDH: Digital DNA–DNA hybridization.
DFU: Diabetic foot ulcer.
ESBL: Extended-spectrum β-lactamase.
EUCAST: European Committee on Antimicrobial Susceptibility Testing.
GN ID: Gram-negative identification.
ICE: Integrative conjugative element.
IS: Insertion sequence.
kb: Kilobase.
kDa: Kilodalton (only if used elsewhere; optional here).
L50: Contig number containing 50% of assembly length.
MDR: Multidrug-resistant.
MIC: Minimum inhibitory concentration.
MGE: Mobile genetic element.
MLST: Multilocus sequence typing.
MOB: Mobilization (relaxase type; e.g., MOBH).
MRS / mcr: Mobilized colistin resistance gene.
NDARO: National Database of Antibiotic Resistant Organisms.
NCBI: National Center for Biotechnology Information.
N50: Contig length metric (50% genome assembly metric).
PGAP: Prokaryotic Genome Annotation Pipeline.
QRDR: Quinolone resistance-determining region.
RNA: Ribonucleic acid.
SNP: Single nucleotide polymorphism.
SPAdes/Unicycler (if needed): genome assembly tools (optional abbreviation).
ST: Sequence type.
tRNA: Transfer RNA.
VITEK: Automated microbial identification system.
WGS: Whole genome sequencing.
